# 2752. Outcomes of Patients with *Stenotrophomonas maltophilia* infections treated with Cefiderocol in PROVE (Retrospective Cefiderocol Chart Review) Study

**DOI:** 10.1093/ofid/ofad500.2363

**Published:** 2023-11-27

**Authors:** Stephen Marcella, Bruce Friedman, Bin Cai, Stefano Verardi, Laurence Gozalo, Sean T Nguyen

**Affiliations:** Shionogi Inc, Florham Park, New Jersey; Joseph M. Still Burn Center, Augusta, Georgia; Shionogi Inc, Florham Park, New Jersey; Shionogi, B.V., London, England, United Kingdom; Genesis Research, Hoboken, New Jersey; Shionogi Inc., Florham Park, New Jersey

## Abstract

**Background:**

Gram-negative (GN) bacterial resistance remains an urgent global health problem. Cefiderocol (CFDC) has activity against *Stenotrophomonas maltophilia* (SM) isolates. PROVE is an ongoing international, retrospective study of CFDC use in GN infections (GNI). This report gives the first description of patients infected with SM as the primary infection treated with CFDC.

**Methods:**

Eligible patients received ≥ 72 hours of CFDC during routine clinical practice attributed to a primary SM infection. Patient characteristics, hospital course, and treatment patterns are described. Outcomes of clinical cure and 30-day all-cause mortality (ACM) were examined by infection site, pathogen phenotype, patient characteristics, and treatment patterns. Clinical cure is defined as resolution or improvement in infection signs and symptoms without later relapse.

**Results:**

As of March 2023, 54 patients were treated with CFDC for a primary SM infection at 39 sites in the US and Europe. The median age was 59 years; 72.2% were male. Chronic pulmonary disease (27.8%), moderate or severe renal disease (18.5%), and COVID-19 (14.8%) were the most frequent comorbidities. Respiratory infections were most common (n=38, 70.4%). 72.2% of patients were given CFDC in an ICU setting. Mechanical ventilation and vasopressor support were required in 57.4% and 40.7%, respectively.

31 (57.4%) of SM infections were polymicrobial GNI, 21 (67.7%) being *Pseudomonas aeruginosa* (Table 1). No SM culture was resistant to CFDC when tested (N=15). The median time from positive culture sample to CFDC therapy was 6 days (interquartile range 4–10). Most (68.5%) of patients had the first dose of CFDC 5 days or more after the index culture was taken (Table 1). CFDC was used as monotherapy in 42.6%. Targeted use with or without preceding failure of other GN antibiotics was 88.9%.

Clinical cure and 30-day ACM were 64.8% and 18.5%, respectively. Monomicrobial and polymicrobial infections had similar clinical cure rates (65.2% and 64.5%), but 30-day ACM was greater for monomicrobial infections (30.4% vs. 9.7%) (Table 1).

**Figure:**
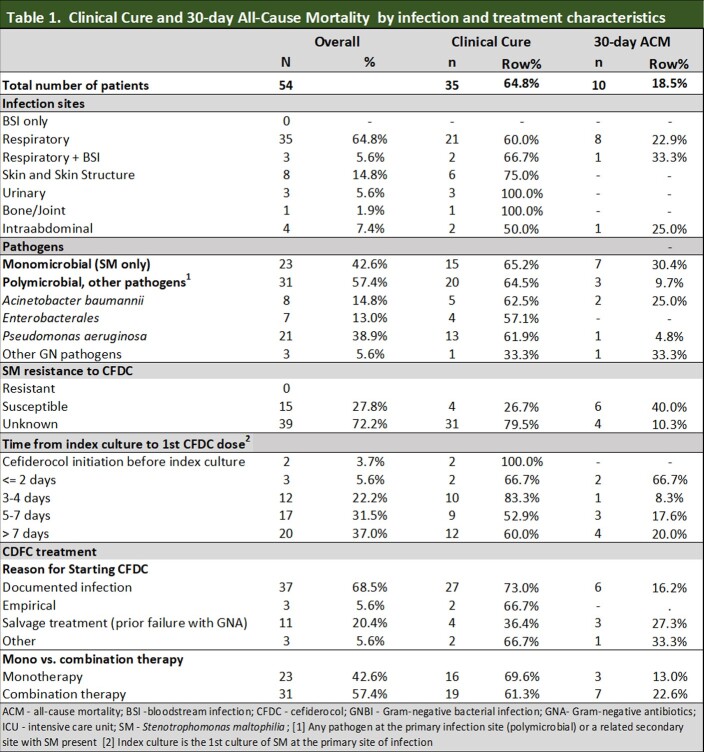

**Conclusion:**

Patients with SM treated with CFDC in a real-world setting had mostly respiratory infections. Over half of SM infections were polymicrobial but had similar rates of clinical cure as those that were monomicrobial.

**Disclosures:**

**Stephen Marcella, MD, MPH**, Shionogi, Inc: contracting work for Shionogi, Inc **Bruce Friedman, MD**, Shionogi: Honoraria **Bin Cai, MD, PhD**, Shionogi Inc.: Shionogi employee **Stefano Verardi, MD**, Shionogi B.V.: Employee **Laurence Gozalo, PhD**, Shionogi, Inc: Analyst **Sean T. Nguyen, PharmD**, Shionogi: Employee|Shionogi, Inc: Employee

